# The greater wax moth, *Galleria mellonella* (L.) uses two different sensory modalities to evaluate the suitability of potential oviposition sites

**DOI:** 10.1038/s41598-022-26826-3

**Published:** 2023-01-05

**Authors:** Saravan Kumar Parepely, Vivek Kempraj, Divija Sanganahalli Dharanesh, Gandham Krishnarao, Kamala Jayanthi Pagadala Damodaram

**Affiliations:** 1 Crop Protection Division, ICAR-Indian Institute of Horticultural Research, Bengaluru, India; 2 Department of Biochemistry, Jain University, Bengaluru, India; 3 USDA-ARS, Daniel K. Inouye Pacific Basin Agricultural Research Center, Hilo, HI USA

**Keywords:** Ecology, Behavioural ecology

## Abstract

An ovipositing insect evaluates the benefits and risks associated with the selection of an oviposition site for optimizing the fitness and survival of its offspring. The greater wax moth, *Galleria mellonella* (L.), uses beehives as an oviposition site. During egg-laying, the gravid wax moth confronts two kinds of risks, namely, bees and conspecific larvae. While bees are known to attack the moth’s offspring and remove them from the hive, the conspecific larvae compete for resources with the new offspring. To date, little is known about the mechanisms involved in the assessment of oviposition site by the greater wax moth, *G. mellonella* (L.). Here, we demonstrate that the wax moth uses two different sensory modalities to detect risks to its offspring in the hives of *Apis cerena*. Bees appear to be detected by the contact-chemoreception system of the gravid wax moth, while detection of conspecifics relies on the olfactory system. Hence, our findings suggest that two different sensory modalities are used to detect two different risks to the offspring and that the selection of oviposition sites by *G. mellonella* (L.) relies on the integration of inputs from both the olfactory and contact-chemoreception systems.

## Introduction

Risk evaluation of an oviposition site is crucial for the fitness of an organism, as choosing inappropriate sites can place the offspring at risk or reduce the offspring’s performance^1–4^. An ovipositing organism is faced with a plethora of challenges and may choose an appropriate oviposition site to avoid predators, competition, or other risks^5–7^. However, oviposition site selection requires an animal to evaluate multiple, possibly, conflicting sensory signals associated with risks and benefits. For example, when a gravid female of *Plutella xylostella* (L.) detects natural enemies (risk) at an oviposition site in their preferred host, the moth tends to chooses an alternate host for oviposition despite knowing that its offspring may develop poorly in the alternate host. The study reveals that herbivorous insects must evaluate both, the risk of natural enemies and the quality of an oviposition site and for a gravid female, the survival of its offspring is crucial than the nutritional quality of the oviposition site^8^. Hence, oviposition site selection behavior provides an excellent means to evaluate how animals perceive and rank various risks.

The greater wax moth, *Galleria mellonella* (L.) (Lepidoptera: Pyralidae) is a noxious pest of honeybees^9^.The moth prefers bee colonies and uses beehive volatiles as cues to locate suitable oviposition sites^9–11^. After locating a suitable beehive from a distance, the gravid female approaches the beehive at night, when the bees are less active, and lays eggs into crevices in the beehive^12^. Hatched neonate larvae of *G. mellonella* tunnel into the honeycomb and feed on pollen, honey, wax, and occasionally brood^9,13,14^. However, the bees do not tolerate the intrusion and eliminate the larvae from the hives^15^. This places the wax moth’s offspring in jeopardy thus making live beehives a less likely site for oviposition. But previous studies have shown that the wax moths are attracted to live beehives and lay eggs in them^10^. A recent study has shown that during oviposition, *G. mellonella* are aware and can detect bee alarm pheromones (isopentyl acetate, benzyl acetate, octyl acetate, and 2-heptanone) but ignores them in favor of an appropriate oviposition site^15^. However, with imminent risk to their offspring does the greater wax moth choose live beehives as a suitable oviposition site when given a choice?

Here, using the interaction between *G. mellonella* (pest) and *Apis cerana* (host), we investigated the risk evaluating behavior during oviposition site selection. We hypothesized that the greater wax moths consider bees and conspecifics as an imminent risk to their offspring and reduce egg-laying in beehives with bees or conspecifics. First, using natural infestation of wax moths to beehives with bees (BB; a known risk to the gravid moth and its offspring), beehives without bees (BW; not a risk to the gravid moth or to its offspring) and beehives with conspecifics (BC; a risk to its offspring), we wanted to see if the wax moths could distinguish the presented risks. Next, we measured the ability of *G. mellonella*’s olfactory system in distinguishing oviposition sites with possible risks using volatile cues from BB, BW and BC. Lastly, we measured the ability of *G. mellonella*’s contact-chemoreception system in distinguishing volatile cues from BB, BW and BC and making egg-laying choice. This interaction of bees and the greater wax moth provides us with an opportunity to understand how organisms integrate inputs from the olfactory and contact-chemoreception systems to detect risk during oviposition site selection.

## Results

### Gravid female wax moths avoid ovipositing in beehives with bees or conspecifics

**Figure 1:**
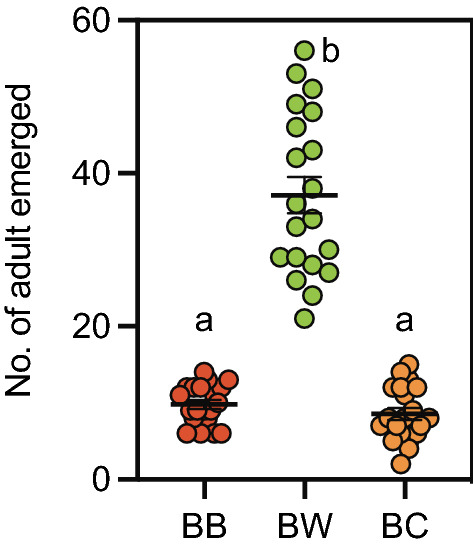
**Field preference of gravid wax moths to beehives**. Gravid female wax moths preferred and laid significantly more eggs in the beehive without bees (BW) than onto beehives with bees (BB) or with conspecifics. Error bars represent s.e.m. Statistical difference was analyzed by one-way ANOVA. Letters indicate statistical differences derived from One-way ANOVA analysis and Tukey’s multiple comparison post hoc tests.

To test gravid female wax moths’ ability to detect bees and conspecific larvae, their attraction and oviposition preferences for beehive with bees (BB), beehive without bees (BW) or beehive with conspecifics (BC) were assessed in the field. Significantly greater moth emergence from BW combs (37.15 ± 2.379 moths; mean ± s.e.m, Tukey’s post hoc *P* < 0.0001) over BB (9.80 ± 0.578 moths) and BC (8.55 ± 0.799 moths) demonstrate the wax moth’s preference for BW (One-way ANOVA; *F*_(2,57)_ = 79.70; *df* = 19; *P* < 0.0001; Fig. 1). This shows the wax moths’ ability to detect and avoid possible risks to their offspring during oviposition site selection.

### Antennal and tarsus responses to chemical cues from BB, BW and BC

**Figure 2:**
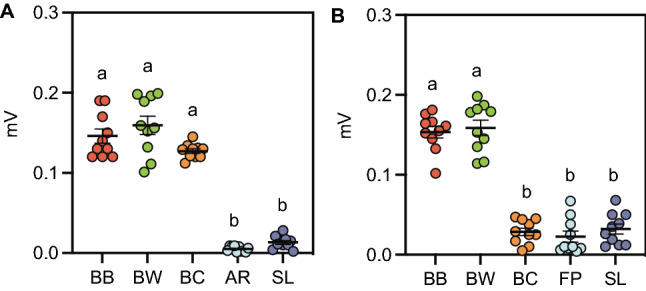
**Electroantennogram (EAG) and Electrotarsogram (ETG) of chemical cues from beehives**. Wax moths’ antenna and tarsus were subjected to electrophysiological study. (**A**) We found that the olfactory system (represented by antenna) responded significantly to chemical cues from beehive with bees (BB), beehive without bees (BW) and beehive with conspecifics (BC). (**B**) However, the contact-chemoreception system (represented by tarsi) responded significantly to chemical cues from beehive with bees (BB) and beehive without bees (BW). Air (AR), filter paper (FP) and solvent (SL) were used as control. Error bars represent s.e.m. Date were analyzed using One-way ANOVA. Similar letters indicate the absence of significant differences.

Insects rely on olfaction to locate oviposition sites from a distance, but upon landing on an oviposition site, they use contact-chemoreception to evaluate the suitability of the site for egg-laying^16^. As shown in the field preference assays, wax moths clearly chose BW as the choice of oviposition site. Therefore, we asked if the moth’s olfactory and contact-chemoreception system, represented by the antenna and tarsus respectively, could detect chemical cues from BB, BW and BC. In electrophysiology experiments, the antenna responded with a higher amplitude to chemical cues from BB = 0.146 ± 0.008 mV (mean ± s.e.m), BW = 0.159 ± 0.011 mV and BC = 0.127 ± 0.002 mV that were significantly different from controls (Air = 0.005 ± 0.001 mV; Solvent = 0.013 ± 0.002 mV). However, the tarsus responded with a higher amplitude to chemical cues of BB (0.153 ± 0.007 mV) and BW (0.158 ± 0.009 mV) and were significantly different from the amplitude of BC (0.028 ± 0.004 mV) and controls (Filter paper = 0.022 ± 0.006; Solvent = 0.032 ± 0.006 mV) (One-way ANOVA; *F*_(4,45)_ = 97.64; *df* = 9; *P* < 0.0001; Fig. 2A,B; Fig. S2.A and B). These results suggest that the wax moth used their olfactory system to detect chemical cues from BB, BW and BC, whereas the contact-chemoreception system could detect cues from BB and BW only.

### Olfactory preference of gravid wax moth to chemical cues from BB, BW and BC

**Figure 3:**
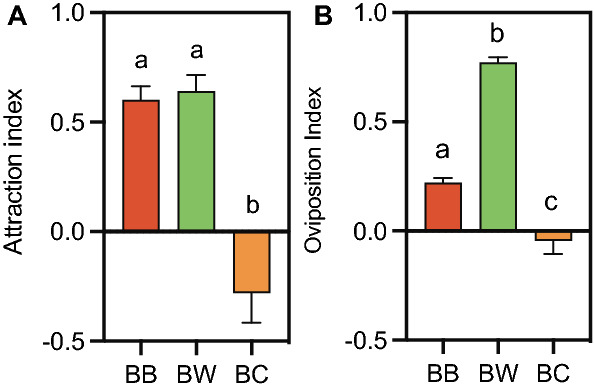
**Attraction and oviposition behavior of wax moths to chemical cues of beehives**. (**A**) In y-tube olfactometer assays, we showed that gravid female wax moths were significantly attracted to chemical cues of beehive with bees (BB) and beehive without bees (BW). However, the chemical cues of beehive with conspecifics (BC) were less attractive to moths. (**B**) Similarly, in oviposition assays, gravid moths preferred to lay significantly more eggs on filter paper with chemical cues of beehive without bees (BW) than on filter paper with chemical cues of beehive with bees (BB) or beehive with conspecifics (BC). Error bars represent s.e.m. Significant difference was analyzed by One-way ANOVA. Similar letter significs that the means are not significantly different.

In field preference assays, wax moths clearly preferred BW as an appropriate oviposition site, suggesting that wax moths were capable of sensing risk to their offspring. Further, electrophysiology studies revealed that the wax moth’s antenna and tarsus responded to chemical cues from BB, BW and BC. However, we wanted to understand the wax moth’s behavior towards these chemical cues. The attraction of gravid female wax moths was measured in Y-tube olfactometer assays to determine their ability to detect the presence of bees or conspecifics from distance using their sense of smell. Our results indicate that female moths were equally attracted (Tukey’s post hoc, *P* = 0.8659) to chemical cues from BB (Attraction index (AI) =  + 0.69 ± 0.049,) and BW (AI =  + 0.68 ± 0.018,), but were less attracted to chemical cues from BC (AI = -0.26 ± 0.047). ANOVA followed by Tukey’s multiple comparison test showed that there was no significant difference between the AI of BB and BW (*P* = 0.8659), but AI of BC was significantly different from AI of BB (*P* < 0.0001) and BW (*P* < 0.0001) (One-way ANOVA; *F*_(2,12)_ = 179.4; *df* = 4; *P* < 0.0001; Fig. 3A). A one sample *t*-test proved that the mean of BB (*t* = 9.487, *df* = 4, *P* = 0.001) and BW (*t* = 8.552, *df* = 4, *P* = 0.001) were significantly different from the theoretical mean of 0, whereas mean of BC (*t* = 2.064, *df* = 4, *P* = 0.108) was not significantly different from 0. From the results, we infer that although the wax moths sense the presence of bees in the volatiles of BB^15^, they still showed equal attraction to the chemical cues of both BB and BW using their olfactory system. However, the moths could detect conspecifics as they avoided the olfactometer arm with chemical cues from BC.

### Oviposition preference of gravid wax moth to headspace volatiles of BB, BW and BC

Next, we assessed the oviposition preference of *G. mellonella* to headspace volatiles of BB, BW and BC using oviposition assays. The moths had access to the filter papers with chemical cues and had no restriction in choosing either the filter paper containing the test samples or control to lay eggs. Gravid moths deposited more eggs on filter paper containing chemical cues of BW with an oviposition index (OI) of 0.77 ± 0.023 (mean ± s.e.m) but deposited less eggs on filter paper with BB (OI = 0.22 ± 0.022) and BC (OI = -0.04 ± 0.059). A one sample *t*-test proved that the mean of BB (*t* = 9.818, *df* = 14, *P* < 0.0001) and BW (*t* = 32.81, *df* = 14, *P* < 0.0001) were significantly different from the theoretical mean of 0, whereas mean of BC (*t* = 0.777, *df* = 14, *P* = 0.450) was not significantly different from 0. One sample *t*-test along with ANOVA followed by Tukey’s multiple comparison test (One-way ANOVA; *F*_(2,42)_ = 127.3; *df* = 14; *P* < 0.0001; Fig. 3B) suggested that *G. mellonella* significantly preferred to lay more eggs onto filter paper with chemical cues of BW than with BB or BC.

### GC–MS analysis of chemical cues from BB, BW and BC

**Figure 4:**
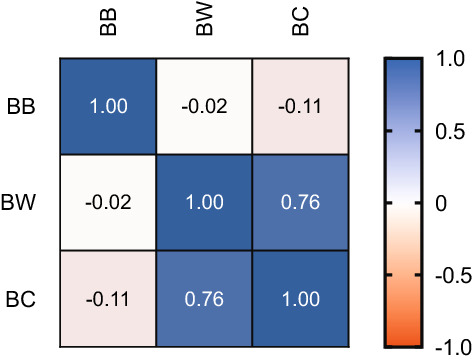
**Multivariant correlation analysis of chemical compounds and their concentrations from the air-entrained chemical cues of beehive with bees (BB), beehive without bees (BW) and beehive with conspecifics (BC)**. The analysis found that the chemical compounds and their concentrations from BW and BC were significantly similar.

GC–MS analysis revealed that the headspace samples from BB, BW and BC contained acids, esters, alkanes, alcohols, aldehydes, and terpenes (Table 1). The similarity of the chemical cues emanating from each source (BB, BW and BC), was examined using a multivariant correlation analysis based on the presence and concentration of compounds. The results suggest that the compounds present in BW and BC and their concentrations were significantly similar (Pearson *r* = 0.76) (Fig. 4), whereas compounds present in BB and their concentrations was different from that of BW (Pearson *r* = − 0.02) and BC (Pearson *r* = − 0.11).

**Table 1:** List of volatile compounds identified from BB, BW and BC using GC–MS.

RT (min)	Compounds	CAS	RI	Class	Mean ± s. e. m (µg/mL)		
					BB	BW	BC
3.91	Methyl valerate*	624-24-8	823	Ester	–	0.45 ± 0.01	13.14 ± 0.07
4.15	Ethyl isovalerate*	108-64-5	854	Ester	–	2.09 ± 0.03	12.53 ± 0.25
6.03	Allyl 2-ethyl butyrate	7493-69-8	995	Ester	–	11.70 ± 0.16	7.71 ± 0.14
6.10	Ethyl hexanoate*	123-66-0	1000	Ester	–	32.94 ± 0.26	26.59 ± 0.47
9.56	Ethyl benzoate*	93-89-0	1171	Ester	121.28 ± 2.65	118.80 ± 2.41	68.29 ± 0.53
10.67	n-Octyl acetate*	112-14-1	1210	Ester	25.40 ± 0.01	–	–
12.37	Benzoic acid, 2,4-dimethyl-, methyl ester	23617-71-2	1295	Ester	11.47 ± 0.17	–	–
13.05	Benzoic acid, 4-ethyl-, methyl ester	7364-20-7	1326	Ester	10.07 ± 0.05	–	–
14.10	(E)-2-Decenyl acetate	2497-23-6	1406	Ester	–	–	92.55 ± 1.35
15.25	Ethyl (E)-cinnamate*	4192-77-2	1463	Ester	–	4.54 ± 0.09	–
16.41	Benzoic acid, 4-ethoxy-, ethyl ester	23676-09-7	1529	Ester	70.08 ± 1.50	–	–
20.75	2-Ethylhexyl salicylate*	118-60-5	1811	Ester	16.91 ± 0.16	–	–
20.98	Isopropyl myristate*	110-27-0	1827	Ester	6.10 ± 0.14	3.69 ± 0.06	15.55 ± 0.10
22.53	Hexadecanoic acid, methyl ester	112-39-0	1926	Ester	–	1.76 ± 0.04	5.84 ± 0.07
22.93	1,2-Benzenedicarboxylic acid, dibutyl ester*	84-74-2	1965	Ester	466.74 ± 0.24	–	–
23.67	Palmitic acid, isopropyl ester*	142-91-6	2023	Ester	16.69 ± 0.20	–	–
4.08	2-Hexanone, 4-methyl	105-42-0	848	Ketone	–	4.68 ± 0.10	–
4.24	Isoamyl methyl ketone*	110-12-3	862	Ketone	–	14.18 ± 0.28	–
4.61	2-Heptanone*	110-43-0	891	Ketone	–	1.39 ± 0.01	–
8.04	2-Nonanone, 3-(hydroxymethyl)-	67801-33-6	1093	Ketone	–	–	42.04 ± 0.28
11.96	4-Ethylacetophenone*	937-30-4	1277	Ketone	214.01 ± 4.57	45.54 ± 0.69	51.88 ± 1.16
12.07	2-Undecanone*	112-12-9	1294	Ketone	–	16.95 ± 0.11	33.23 ± 0.10
14.35	trans-α-Ionone*	127-41-3	1426	Ketone	1.09 ± 0.03	–	–
14.78	1,4-Diacetylbenzene*	1009-61-6	1461	Ketone	78.34 ± 0.20	–	–
11.52	2-(2-Butynyl) cyclohexanone	54166-48-2	1267	Ketone	56.84 ± 1.18	–	–
22.21	2-Heptadecanone*	2922-51-2	1902	Ketone	–	–	11.27 ± 0.16
4.39	2,2,4-Trimethyl-3-pentanol*	5162-48-1	882	Alcohol	–	–	13.42 ± 0.32
5.10	4-Methylcyclohexanol*	589-91-3	928	Alcohol	226.04 ± 5.06	–	–
8.55	Phenylethyl Alcohol*	60-12-8	1116	Alcohol	–	–	78.19 ± 1.99
8.63	2-Cyclohexen-1-ol, 1-methyl-4-(1-methylethenyl)-, trans	7212-40-0	1123	Alcohol	–	5.65 ± 0.06	–
9.96	2,3-Xylenol*	526-75-0	1180	Alcohol	11.64 ± 0.21	–	–
10.09	p-Cymen-8-ol*	1197-01-9	1183	Alcohol	4.42 ± 0.10	–	–
15.91	Epicubebol	38230-60-3	1493	Alcohol	–	3.90 ± 0.07	–
16.36	Cubebol	23445-02-5	1515	Alcohol	–	7.67 ± 0.02	–
16.92	α-Cedrol	77-53-2	1598	Alcohol	46.07 ± 0.41	3.65 ± 0.05	–
19.14	2-Hexadecanol*	14852-31-4	1702	Alcohol	45.77 ± 0.62	0.23 ± 0.00	15.38 ± 0.02
21.95	Cedran-diol, (8S,14)-	62600-05-9	1876	Alcohol	14.98 ± 0.13	–	–
22.39	2-Heptadecanol	16813-18-6	1909	Alcohol	139.30 ± 3.12	–	–
4.88	Heptanal*	111-71-7	901	Aldehyde	65.82 ± 0.25	–	–
8.32	Nonanal*	124-19-6	1104	Aldehyde	45.57 ± 1.00	35.30 ± 0.57	–
9.81	Ethyl-benzaldehyde*	4748-78-1	1180	Aldehyde	58.40 ± 1.43	25.25 ± 0.47	8.05 ± 0.09
10.34	Decanal*	112-31-2	1206	Aldehyde	65.60 ± 1.47	12.36 ± 0.03	7.89 ± 0.10
5.65	Phenol*	108-95-2	980	Phenol	–	20.27 ± 0.24	98.15 ± 1.13
9.16	o-Ethylphenol*	90-00-6	1139	phenol	–	10.31 ± 0.15	47.96 ± 0.22
12.74	2-Allyl-4-methylphenol*	6628-0-64	1316	Phenol	3.00 ± 0.01	–	–
14.07	Methyleugenol*	93-15-2	1402	Phenol	65.70 ± 0.14	4.81 ± 0.05	–
16.26	Phenol, 2,5-bis(1,1-dimethylethyl)	5875-45-6	1514	Phenol	0.84 ± 0.02	0.00 ± 0.00	–
5.89	Hexanoic acid*	142-62-1	990	Fatty acid	23.91 ± 0.26	21.25 ± 0.40	–
7.65	Heptanoic acid*	111-14-8	1078	Fatty acid	90.44 ± 0.00	45.050.35	13.00 ± 0.00
11.60	Nonanoic acid*	112-05-0	1273	Fatty acid	180.65 ± 1.50	–	–
13.58	4-Ethylbenzoic acid*	619-64-7	1363	Fatty acid	87.48 ± 1.82	–	–
13.85	3,4-Dimethylbenzoic acid	619-04-5	1387	Fatty acid	58.94 ± 0.28	–	–
16.12	2,4,6-Trimethylmandelic acid	20797-56-2	1504	Fatty acid	27.15 ± 0.41	11.05 ± 0.14	–
22.76	Palmitoleic acid*	373-49-9	1951	Fatty acid	–	3.69 ± 0.03	–
23.00	n-Hexadecanoic acid*	57-10-3	1968	Fatty acid	–	1.40 ± 0.00	11.27 ± 0.18
6.24	psi-limonene*	499-97-8	1004	Terpene	–	82.08 ± 1.92	125.82 ± 2.18
6.37	3-Carene*	13466-78-9	1011	Terpene	21.35 ± 0.29	24.28 ± 0.21	–
6.87	Z-β-Ocimene*	13877-91-3	1037	Terpene	–	121.50 ± 2.85	232.15 ± 1.21
7.54	E-β-Ocimene*	3779-61-1	1049	Terpene	5.33 ± 0.12	228.26 ± 3.09	309.96 ± 3.87
8.80	Allo-Ocimene*	673-84-7	1131	Terpene	–	100.99 ± 0.42	104.26 ± 0.22
9.08	2,4,6-Octatriene, 2,6-dimethyl-,(E,Z)*	7216-56-0	1131	Terpene	–	22.32 ± 0.28	23.57 ± 0.27
12.17	Indole*	120-72-9	1295	Terpene	–	26.96 ± 0.39	107.10 ± 0.22
13.62	Cyclosativene	22469-52-9	1368	Terpene	–	13.67 ± 0.01	–
13.76	α-Copaene	3856-25-5	1376	Terpene	–	17.63 ± 0.27	–
13.79	Di-epi-α-cedrene	50894-66-1	1384	Terpene	–	56.82 ± 0.95	16.16 ± 0.29
6.66	o-Cymene*	527-84-4	1023	Aro. HC	–	35.43 ± 0.84	36.60 ± 0.58
6.73	p-Cymene*	99-87-6	1025	Aro. HC	22.61 ± 0.56	96.95 ± 2.42	84.68 ± 1.15
8.54	Benzene, 1,2,4,5-tetramethyl*	95-93-2	1116	Aro. HC	–	5.62 ± 0.00	–
12.67	1H-Indene, 1-ethylidene	2471-83-2	1315	Aro. HC	–	32.00 ± 0.30	–
13.96	3,5-Heptadienal, 2-ethylidene-6-methyl-	99172-18-6	1395	Aro. HC	–	–	34.81 ± 0.29
14.66	Hexamethylbenzene*	87-85-4	1434	Aro. HC	–	6.95 ± 0.03	29.01 ± 0.11
16.23	Benzene, 1,4-dimethoxy-2,3,5,6-tetramethyl	13199-54-7	1511	Aro. HC	–	–	17.41 ± 0.20
7.07	γ-Vinyl-γ-valerolactone	1073-11-6	1043	Lactone	27.27 ± 0.45	–	–
11.42	γ-Octalactone*	104-50-7	1261	Lactone	–	63.18 ± 1.51	16.93 ± 1.31
24.95	γ-Palmitolactone	730-46-1	2105	Lactone	8.35 ± 0.16	–	–
8.21	n-Undecane*	1120-21-4	1100	Hydrocarbon	45.69 ± 0.55	43.54 ± 0.78	56.72 ± 1.33
10.19	n-Dodecane*	112-40-3	1200	Hydrocarbon	–	36.27 ± 0.49	152.27 ± 2.14
12.53	n-Tridecane*	629-50-5	1300	Hydrocarbon	14.97 ± 0.29	–	17.41 ± 0.16
14.04	n-Tetradecane*	629-59-4	1400	Hydrocarbon	–	19.56 ± 0.42	26.24 ± 0.40
16.05	n-Pentadecane*	629-62-9	1500	Hydrocarbon	40.11 ± 0.61	–	–
16.48	2,6,10-Trimethyltetradecane	14905-56-7	1539	Hydrocarbon	39.12 ± 0.83	2.37 ± 0.02	19.68 ± 0.02
17.38	n-Hexadecane*	544-76-3	1600	Hydrocarbon	11.30 ± 0.06	–	–
18.77	2-Methylhexadec-1-ene	61868-19-7	1687	Hydrocarbon	3.53 ± 0.04	–	–
19.03	n-Heptadecane*	629-78-7	1700	Hydrocarbon	19.63 ± 0.04	14.31 ± 0.08	28.83 ± 0.32
20.05	Phytane*	638-36-8	1792	Hydrocarbon	32.22 ± 0.22	–	–
20.50	Crocetane*	504-44-9	1792	Hydrocarbon	9.48 ± 0.03	–	–
22.15	n-Nonadecane*	629-92-5	1900	Hydrocarbon	–	–	12.65 ± 0.17
23.55	n-Eicosane*	112-95-8	2000	Hydrocarbon	16.72 ± 0.27	–	–
23.95	10-Methylicosane	54833-23-7	2042	Hydrocarbon	11.41 ± 0.13	–	–
24.65	2-Methylicosane	1560-84-5	2063	Hydrocarbon	9.48 ± 0.05	–	–
24.92	n-Heneicosane*	629-94-7	2100	Hydrocarbon	–	0.96 ± 0.01	18.95 ± 0.45
10.83	exo-2-Hydroxycineole	92999-78-5	1224	Monoterpenoids	–	11.69 ± 0.03	23.94 ± 0.20
14.21	beta-caryophyllene*	87-44-5	1419	Sesquiterpenes	65.04 ± 0.43	6.56 ± 0.07	0.67 ± 0.02
15.33	γ-Selinene	515-17-3	1481	Sesquiterpenes	17.42 ± 0.38	3.82 ± 0.08	6.73 ± 0.05
15.50	Aristolochene	26620-71-3	1487	Sesquiterpenes	–	132.01 ± 0.00	–
18.35	Cedryl propyl ether	19870-75-8	1652	Ether	10.09 ± 0.05	–	–
15.73	3-Buten-2-one, 4-(2,2,3-trimethyl-6-methylenecyclohexyl)-	79-68-5	1490	Others	25.05 ± 0.38	–	–
16.61	2-Heptanone, 6-(3-acetyl-2-methyl-1-cyclopropen-1-yl)-6-methyl-	65868-86-2	1565	Others	7.78 ± 0.15	–	–

*RT (min)* retention time in minutes, *RI* retention index, *BB* beehive with bees, *BW* beehive without bees, *BC* beehives with conspecific, *s.e.m* Standard error mean, *Compound identifications were verified using commercial synthetic standards (purchased from Sigma-Aldrich, India), *Aro. HC* aromatic hydrocarbon.

## Discussion

The oviposition strategy of an insect is a complex process with trade-off between many factors^17–19^. Risk to an insect’s offspring is one factor that can have a strong influence on its oviposition site selection^1–5,20–22^. A mated female insect may select a specific oviposition site to avoid predators or competitors, or because the site has advantageous physical characteristics^2,7,23,24^. Discriminating risks at an oviposition site is challenging and a costly affair, it is therefore crucial for a gravid insect to detect specific chemical information from a distance to distinguish suitable from unsuitable oviposition sites^6,25–30^. Here, we demonstrate that the gravid wax moths could detect risks at the oviposition sites by integrating inputs from two separate sensory modalities.

Previous studies revealed that the *G. mellonella* communicates through different sensory modalities, including auditory and pheromone signalling^31^. Wax moths are known to utilize their ears to detect risk auditory signals from echolocating insectivorous bats and decide to tradeoff the signals to find mate and an appropriate oviposition site^32,33^. But as they attack the beehives at night, when the bees are less active, detecting auditory signals from beehives is less likely. Therefore, we considered the olfactory and contact-chemoreception for our studies. In field preference assays, gravid wax moths considered bees and conspecifics as risks, preferring BW over BB and BC as oviposition site. Interestingly, in olfactometer assays, where gravid wax moths were allowed to use only their olfactory system to make choices, gravid moths preferred and were significantly attracted to chemical cues of BB and BW but were not attracted to chemical cues from BC. This proved that the wax moth’s olfactory system could only sense the risk posed by conspecifics and not by bees^15^. In oviposition assays, where the moths are allowed to use both their olfactory and contact-chemoreception system to make choices, gravid moths laid significantly less eggs on filter paper with chemical cues from BB and BC. This clearly proved that the moth’s contact-chemoreception system is required to detect bees as a risk. In addition, electrophysiology studies also revealed that the wax moth’s response was higher to BW rather than BB and BC, which gives us a clue that the contact-chemoreceptors situated on the tarsus are tuned to some of the volatile compounds from BW which evoked the oviposition in greater wax moth. Further studies are being conducted to identify these oviposition cues. In a recent study, Kwadha et al.^34^ detected terpenes (sylvestrene), aldehydes (nonanal and decanal), and esters (ethyl propanoate, 2-methyl ethyl propanoate, 2-methyl ethyl butanoate, and 3-methyl butyl acetate) as some of the cues that attract *G. melonella* to honeycomb volatiles. These cues may be significant in mediating oviposition behaviour in the greater wax moth.

Another interesting aspect in this study that requires an in-depth analysis was the wax moth’s choice of oviposition site based on chemical cues. Upon multivariant correlation analysis of the chemical compounds from BB, BW and BC, we found BW and BC to be significantly similar. This meant that the wax moths should have been attracted to BC as much as they did to BW. However, this was not the case either in the field, olfactometer or oviposition assays. This clearly shows that the chemical cues, although important, is not the only criteria in decision making process in insects. It is imperative that an insect can contextually integrate sensory signals to formulate appropriate behavioral responses^35^.

**Figure 5:**
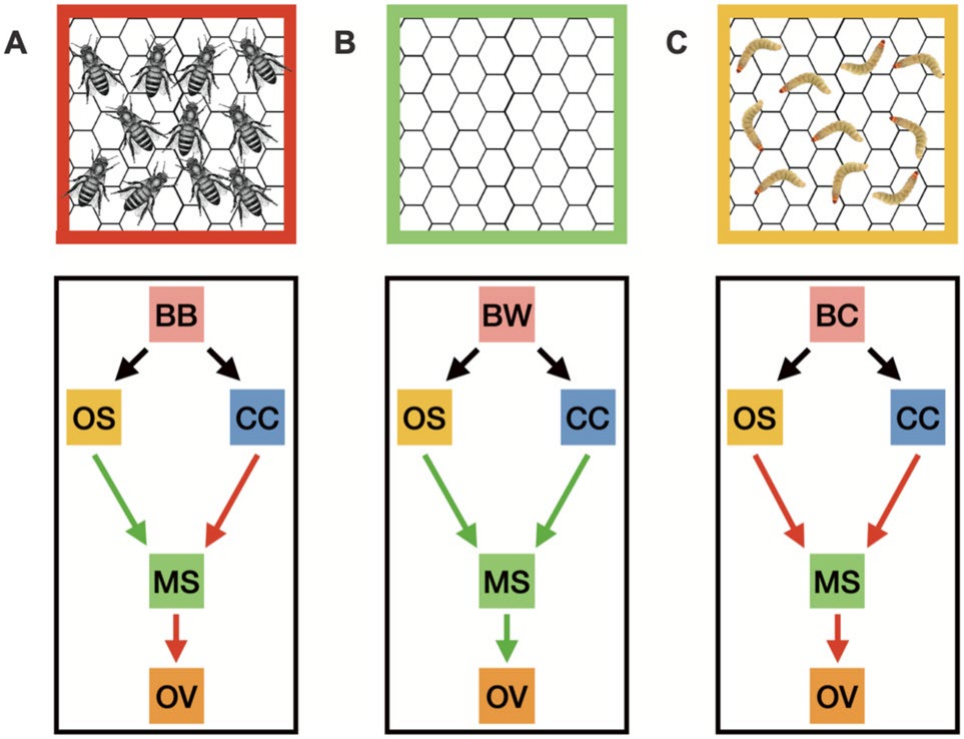
**Neuronal models for the interaction between contact-chemoreception (CC) and olfactory system (OS) of wax moth during oviposition site selection**. (**A**) Model 1: When the moth senses a BB, the positive signal (green arrow) from their olfactory system to the motor system instigates flight towards the beehive, upon landing on the beehive their contact-chemoreception system senses the presence of bees and sends a negative signal (red arrow) to the motor system (MS), thus restraining oviposition (OV). (**B**) Model 2: Similarly, when a moth senses a BW, positive signals sent to the motor system from both the olfactory and contact-chemoreception system instigates flight towards BW and stimulates oviposition. (**C**) Model 3: However, when a moth senses a BC, the olfactory system sends a negative signal to the motor system thus causing no flight towards BC.

Considering the signal integration from olfactory and contact-chemoreception system in the wax moth, we developed three possible neuronal models (Fig. 5). Model 1: When the moth senses a BB, the positive signal from their olfactory system to the motor system instigates flight towards the beehive, upon landing on the beehive their contact-chemoreception system senses the presence of bees and sends a negative signal to the motor system, thus restraining oviposition (Fig. 5A). Model 2: Similarly, when a moth senses a BW, positive signals sent to the motor system from both the olfactory and contact-chemoreception system instigates flight towards BW and stimulates oviposition (Fig. 5B). Model 3: However, when a moth senses a BC, the olfactory system sends a negative signal to the motor system thus causing no flight towards BC (Fig. 5C). Our data clearly supports the neuronal model where central integration of signals from olfactory and contact-chemoreception systems mediate risk detection by female moths. However, further work on the neurobiology of this moth needs to be done to validate these models. We suggest that our paradigm can be used as a simple model for risk detection by ovipositing insects. Apart, our study can also play an important role in improving control methods of this devastating pest of bees.

## Materials and methods

### Insects

*Galleria mellonella* larvae (7–8th instar) and *Apis cerena* colonies were obtained from the Department of Entomology, University of Agricultural Sciences, GKVK, Bengaluru, India and maintained at the Division of Crop Protection, ICAR-Indian Institute of Horticultural Research, Bengaluru, India. Larvae of *G. mellonella* were reared on honeycombs of *A. cerana* in a dark plastic container (20 × 20 × 30.5 cm; length × width × height), at ambient conditions (27 ± 1 °C, 75 ± 2% RH and 14L: 10D h photoperiod). Cocoons (n = 150) of *G. mellonella* were collected and placed in cages (30 × 30 × 30 cm^2^) for emergence. Emerged adult moths were provided with honey solution (2%) and water moistened on cotton swabs ad libitum. Adults, 3–5 days old, were allowed to mate for 48 h and gravid females were separated into different cages for use in further experiments. *A. cerena* colonies in bee boxes were placed in the shade in a mango orchard at ICAR-Indian Institute of Horticultural Research, Bengaluru, India (13.1348° N, 77.4960° E) and were provided with water in a container and were allowed to forage on flowers in the orchard.

### Preference of wax moth to BB, BW and BC in field conditions

Beehive with bees (BB), beehive without bees (BW) and beehive with conspecific damage (BC) were positioned in beehive boxes. These boxes were placed in the field for a week (during peak infestation season, July–August) for the infestation of *G. mellonella* to occur. After the field exposure period of one week, the exposed combs were collected and placed separately in cages (30 × 30 × 30 cm) for adult moths to emerge. For combs of BB, the bees were transferred to a new comb as they may detect larvae and remove them from the beehive. The number of adults emerged from BB, BW and BC were enumerated. A total of 20 trials was conducted for each kind of beehive.

### Headspace volatile collection from BB, BW and BC

Headspace collections from BB, BW and BC were performed at night (7 pm to 7 am Indian Standard Time) using a customized air entrainment system described by Kamala Jayanthi et al*.*^36^. Porapak Q (50 mg; 60/80 mesh; Supelco, Sigma-Aldrich) was packed in a glass tube (L = 5 cm, dia. = 5 mm) and activated at 180 °C under a stream of nitrogen. Activated Porapak Q tubes were used to collect headspace volatiles. A beehive box consisting of a strong healthy bee colony (5–6 months old), a beehive box without bees but with intact honeycombs and a beehive box with honeycombs damaged by wax moth larvae were selected for headspace volatile collection. All connections were made with polytetrafluoroethylene (PTFE) tubing with brass ferrules and fittings (Swagelok, India) and sealed with PTFE tape. The tubes fitted with Porapak Q were gently inserted into an opening in the beehive boxes and air was drawn at the rate of 400 mL/min. Simultaneously, headspace volatiles from an empty bee box were entrained for use as a control. Volatiles was entrained for 12 h and was eluted with 750 µL of redistilled diethyl ether (99.7% pure, Merck, India). The eluted headspace volatile samples were collected in clean glass vials (2 mL; Supelco, India). *n*-Nonyl acetate, (99.9%, Sigma-Aldrich, India), (5 µL; 500 ng/µL) was added to the headspace volatile samples as an internal standard for quantification^37^ and were stored at − 20 °C until further use.

### Y-tube olfactometer assays

A glass Y-tube olfactometer (Fig. S1. A; I.D. = 3.5 cm; length of main stem and side arms = 30 cm; angle of Y = 90°) was used to test the attraction of mated *G. mellonella* to headspace volatiles of beehive with bees (BB), beehive without bees (BW), beehive with conspecific damage (BC) and control. Before each experiment, all glassware was washed with a non-ionic detergent, rinsed with acetone, and distilled water, and dried in an oven at 180 °C for 2 h. The experiment was set up in a dark room, at night (7 pm to 9 pm), at ambient conditions (27 ± 2 °C and 60 ± 5% RH). Test sample (50 µL) and control (50 µL solvent) were dispensed onto separate cotton wicks (3–5 inch) and was allowed 1 min for the solvent to evaporate. The cotton wicks were then placed into two gas wash bottles with an inlet and an outlet. The Y-tube setup aided the air passed through an activated charcoal filter to be pushed separately into the gas wash bottles with one holding a cotton wick with test sample and the other holding a cotton wick with control through the inlet. The air from the outlet of the gas wash bottle loaded with headspace volatiles was gently pushed into the treatment arm and the air from the outlet of the gas wash bottle loaded with control was pushed into the control arm^38^. The flow rate was adjusted to 1.5 mL/min using a flowmeter. Gravid females of *G. mellonella* were introduced individually through an opening in the main stem of the olfactometer (Fig. S1). In a replicate, each moth was given 2 min to acclimatize in the olfactometer, after which the experiment was run for 5 min. Each set of the Y-tube olfactometer assay was repeated 5 times for a test sample, with each set having 10 replicates. If a moth did not choose any arm, the replicate was discarded and repeated. Attraction index (AI) was calculated using the formula AI = [(no. of moth entry into treatment arm – no. of moth entry into control arm)/(total no. of moth entry into treatment arm + no. of moth entry into control arm)]. After each replicate, the direction of the olfactometer was changed to eliminate any directional bias. Each insect was used only once in the assay.

### Oviposition assay

Oviposition assay for *G. mellonella* was conducted in a transparent plastic box (30 cm × 20 cm × 15 cm) divided into two equal parts with one part containing filter paper disc with solvent and the other part containing the filter paper with headspace volatiles of BB, BW,BC and control (50 µL). Test samples or solvent were applied onto a filter paper disc (Whatman No. 1, 5 cm length, 3 cm breadth) using a micropipette and the solvent was allowed to evaporate before placing the filter paper disc inside the plastic box. Gravid females *G. mellonella* were individually released into a plastic box closed with a black muslin cloth. Moths were given 24 h to lay eggs. The assays were conducted at night (7 pm onwards). Eggs laid on the filter paper were enumerated using a Leica M205 series stereomicroscope. Oviposition index (OI) was calculated using the formula OI = [(no. of eggs laid on treatment filter paper – no. of eggs laid on control filter paper) / (no. of total eggs laid)]. A single insect was used per trial, and 15 trials were conducted to test moth oviposition preferences between each test samples and a control.

### Electrophysiological recording

Electrophysiological recordings were done using the antenna (olfaction) and tarsus (contact-chemoreception) of gravid female *G. mellonella* (2–3 days old). The test insect was immobilized by chilling on ice. The antenna or tarsus of the immobilized insect was excised using a pair of micro-scissors and placed on a probe holder (Syntech, Germany, Fig. S1. B) containing a small amount of electrode gel (Signa, Parker Laboratories, USA). The base of the antenna or tarsus was placed touching the indifferent ground electrode and the other end touching the recording electrode. For olfactory stimuli, 10 µL of the headspace volatiles (BB, BW or BC) were pipetted onto filter paper strips (Whatman No. 1, 6 cm length × 0.5 cm breadth). The solvent was allowed to evaporate for 30 s before placing the filter paper inside a glass Pasteur Pipette (10 cm length and 6 mm outer dia.). The probed antenna was stimulated by puffing purified air (continuous airflow of 300 mL/min) carrying headspace volatile samples (for 0.5 s) over the antennae. For contact-chemoreception stimuli, 10 µL of the headspace volatiles (BB, BW or BC) were pipetted onto separate filter paper strips (Whatman No. 1, 6 cm length × 0.5 cm breadth). The stimuli for puffs were random and no specific sequence followed. The solvent was allowed to evaporate for 30 s before fixing the filter paper strip to a clip attached to a micro-manipulator. The probed tarsus was stimulated by carefully touching the filter paper containing headspace volatiles to the tarsus for 1–2 s. The responses from the antenna and tarsus were acquired using an Intelligent Data Acquisition Controller (Syntech Model IDAC-4,) and recorded using AutoSpike software (Syntech, Germany). The configuration in the AutoSpike properties tab for the channel with the electrophysiology probe was set at a sampling rate of 100 and a filter of 0–32 Hz. The responses (amplitudes) to the treatments were expressed as the mean of all recorded antennal depolarizations. A total of 10 replicates were carried out for each treatment, and fresh antenna or tarsus was used for each recording. Filter paper with solvent was used as control.

### Gas chromatography coupled mass spectrometry analysis (GC–MS)

The chemical composition of headspace samples of BB, BW and BC was analyzed using Gas-chromatography (Agilent 7890B GC system) coupled with a mass spectrometer (Agilent 5977 MSD). A capillary column (HP-5 MS UI column of L = 30 m, Dia. = 0.25 mm & Thickness = 0.25 µm) was used to examine samples. The thermal program was set initially at an oven temperature of 60 °C for 1 min, and then ramped at 7 °C/min to 240 °C, with helium as the carrier gas, with the flow rate 1 mL/min and held for 2 min at pressure 8.3 psi. Mass spectrometer was in full scan mode (70 eV) and atomic mass unit (AMU) ranged from 40 to 450. One microliter of the sample was injected in split-less mode (40 mL/min) with injection temperature at 270 °C. Individual compounds were identified using the Kovats Index, calculated using a homologous series of n-alkanes (C_7_ to C_30_; Sigma-Aldrich) as standard^39^, and comparing the MS spectra with a spectral library, NIST 14. Identified compounds were authenticated by co-injecting standard synthetic compounds along with samples. Quantification of volatiles was performed using the internal standard method^37^ and the relative abundance of each compound was calculated based on the internal standard of *n*-nonyl acetate.

### Statistics

Statistical analyses were performed using GraphPad Prism version 9.1 for Mac (GraphPad Software Inc, San Diego, CA, USA). Data were subjected to normal distribution tests before any statistical analysis. One-way ANOVA followed by Tukey’s multiple comparison test was used as data conformed to a normal distribution. Error bars in figures were mean ± standard error of the mean (s.e.m). The means of attraction index (AI) and oviposition index (OI) followed normal distribution, the means were subjected to one sample *t*-test to find if the means were significantly different from 0. The mean of relative abundance of compounds detected from BB, BW and BC were subjected to multivariant correlation analysis. A correlation matrix was constructed to understand the similarity of the volatile sources. The matrix contains the Pearson R value.

## Supplementary Information

Supplementary Legends.

Supplementary Figure S1.

Supplementary Figure S2.

## Data Availability

The datasets generated and analyzed during this current study are available from ResearchGate (https://doi.org/10.13140/RG.2.2.17899.21289).
