# Supplementary material for: The greater wax moth, Galleria mellonella (L.) uses two different sensory modalities to evaluate the suitability of potential oviposition sites
Source: Sci Rep. 2023 Jan 5;13:211. doi: 10.1038/s41598-022-26826-3 (PMC9814581; doi:10.1038/s41598-022-26826-3)
Supplement: Supplementary file 1 — Supplementary Legends. [file 41598_2022_26826_MOESM1_ESM.docx]

**Supplementary Information**

**Figure S1. (A**). A schematic design of a Y-tube olfactometer with two arms, one serving as a treatment arm and the other as the control arm. (**B)**. Electrophysiology preparation for antennae and tarsus.

**Figure S2. (A).** Representative electroantennograms for antennal response (EAG). **(B)**. Representative electrotarsograms for tarsi response (ETG).
