# Supplementary figures and images for: The greater wax moth, Galleria mellonella (L.) uses two different sensory modalities to evaluate the suitability of potential oviposition sites
Source: Sci Rep. 2023 Jan 5;13:211. doi: 10.1038/s41598-022-26826-3 (PMC9814581; doi:10.1038/s41598-022-26826-3)

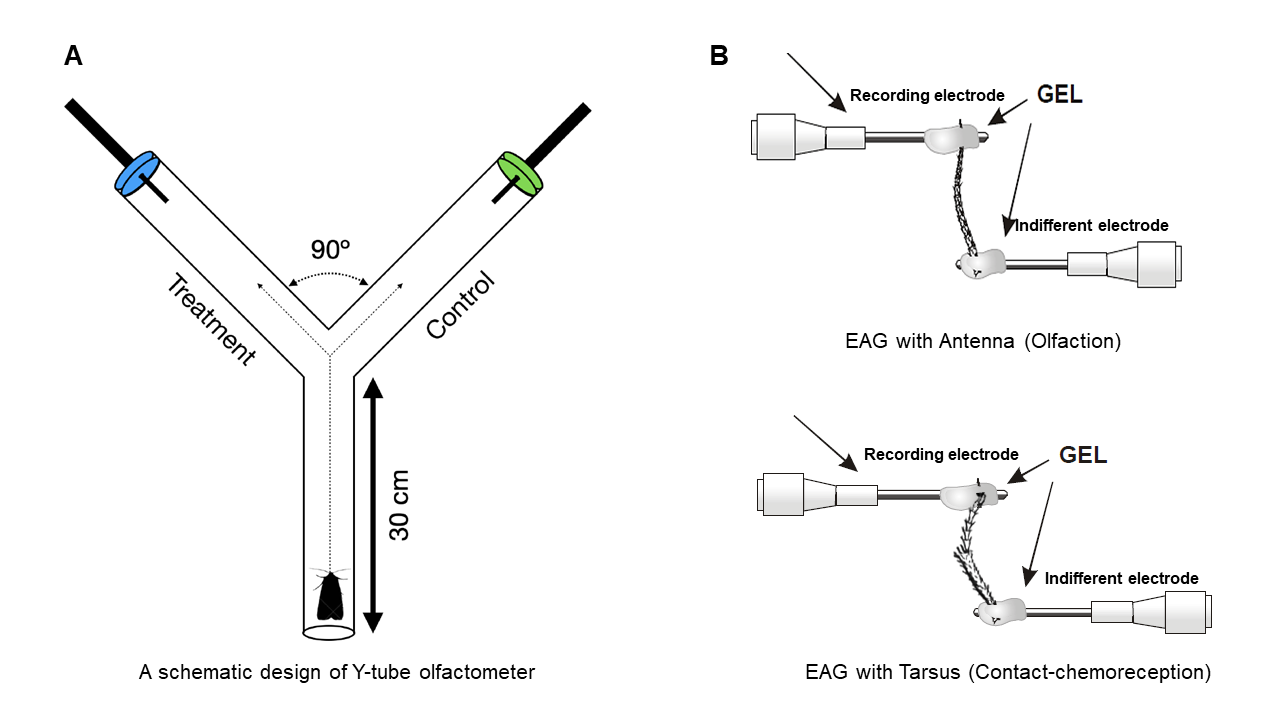

Supplement: Supplementary file 2 — Supplementary Figure S1. [file 41598_2022_26826_MOESM2_ESM.tif]

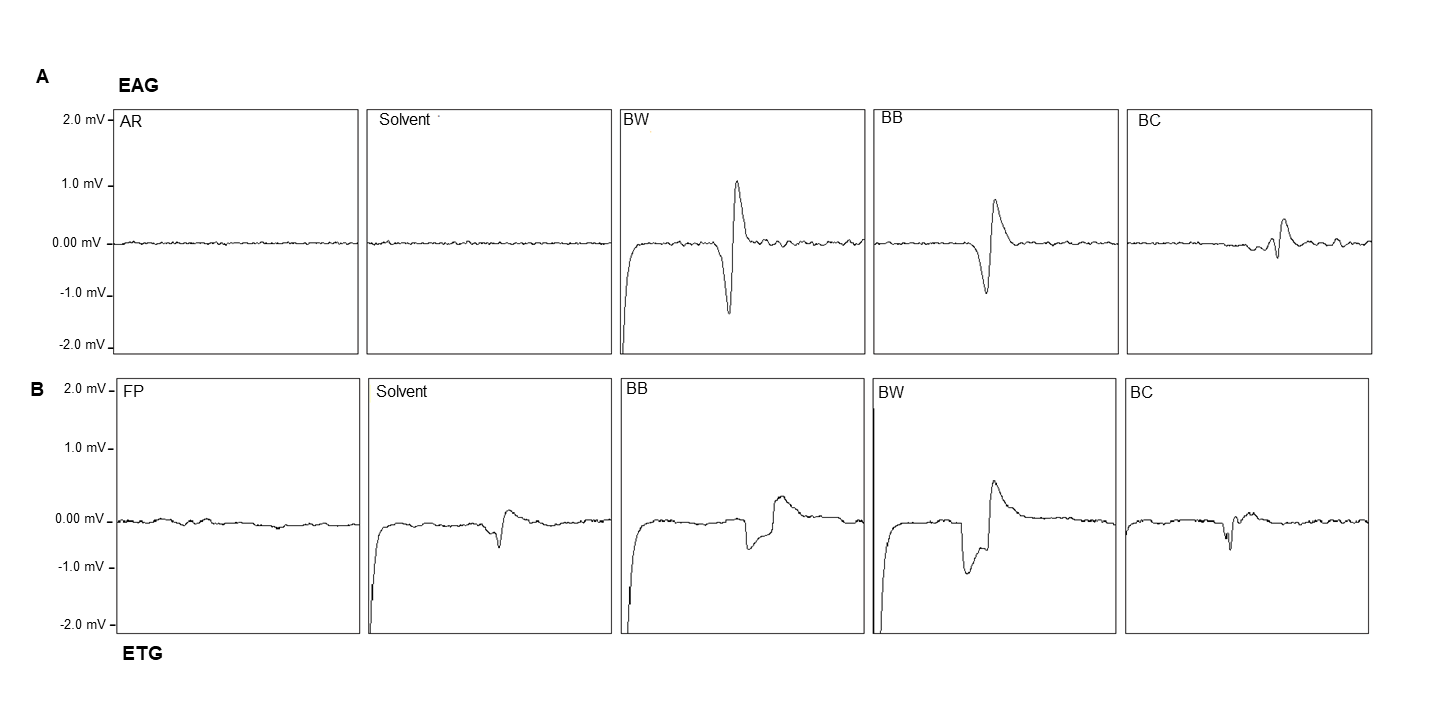

Supplement: Supplementary file 3 — Supplementary Figure S2. [file 41598_2022_26826_MOESM3_ESM.tif]
